# Supplementary material for: A Comprehensive Profiling of Cellular Sphingolipids in Mammalian Endothelial and Microglial Cells Cultured in Normal and High-Glucose Conditions
Source: Cells. 2022 Sep 30;11(19):3082. doi: 10.3390/cells11193082 (PMC9563724; doi:10.3390/cells11193082)
Supplement: Supplementary file 1 [file cells-11-03082-s001.zip › cells-1848924-supplementary.pdf]

A Comprehensive Profiling of Cellular Sphingolipids in Mammalian Endothelial and Microglial Cells Cultured in Normal and High-Glucose Conditions

Figure S1:

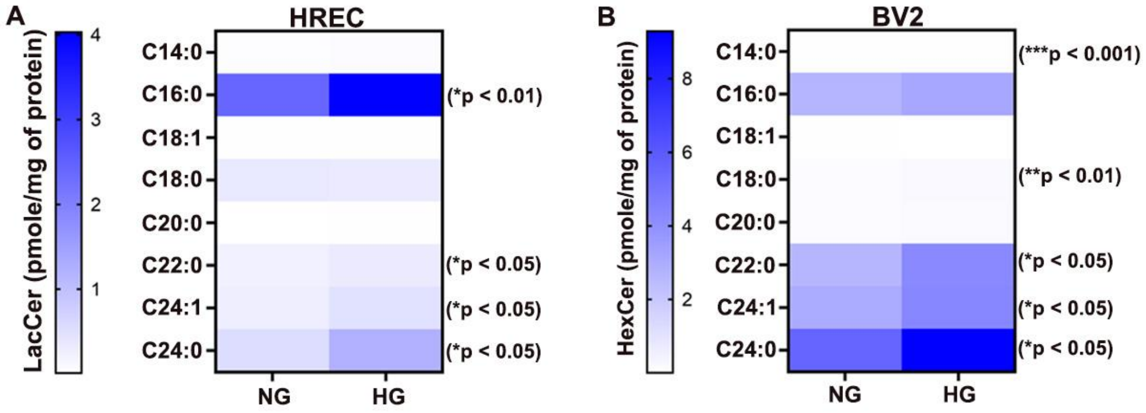

Figure S1: Heat map of Glycosphingolipids in high glucose-cultured HREC and BV2 cells. Different carbon chains of Lactosyl-Ceramide (LacCer) (pmole/mg protein) in Normal glucose (NG) and High glucose (HG)-cultured HREC (A). Different carbon chains of Hexosyl-Ceramide (HexCer) (pmole/mg protein) in NG and HG-cultured BV2 (B). $^*p < 0.05$, $^{**}p < 0.01$, $^{***}p < 0.001$, Mean ± SEM, n=6.

Figure S2:

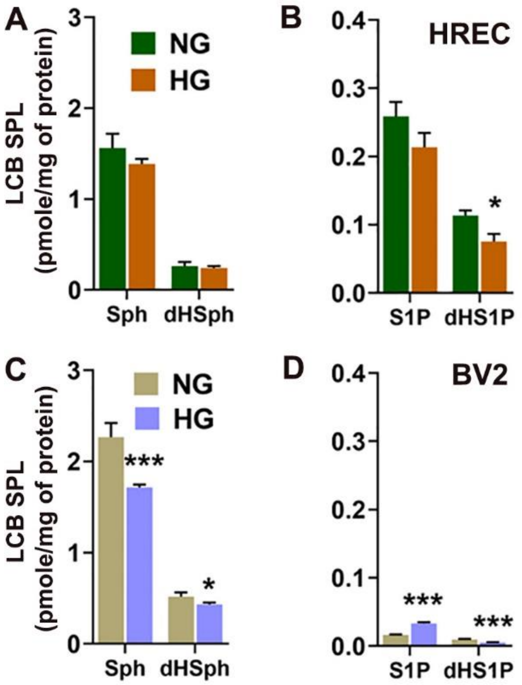

Figure S2: Analysis of different Long Chain Base (LCB) Sphingolipid (SPL) classes in high glucose cultured HREC and BV2 cells. Levels of LCB-SPL (Sphingosine, Sph; dihydrosphingosine, dHSph; sphingosine-1-phosphate, S1P and dihydro S1P, dHS1P) (pmole/mg protein) in Normal Glucose (NG) and High glucose (HG)-cultured HREC (A, B) and BV2 (C, D). *$p < 0.05$, ***$p < 0.001$, Mean ± SEM, n=6.

Figure S3:

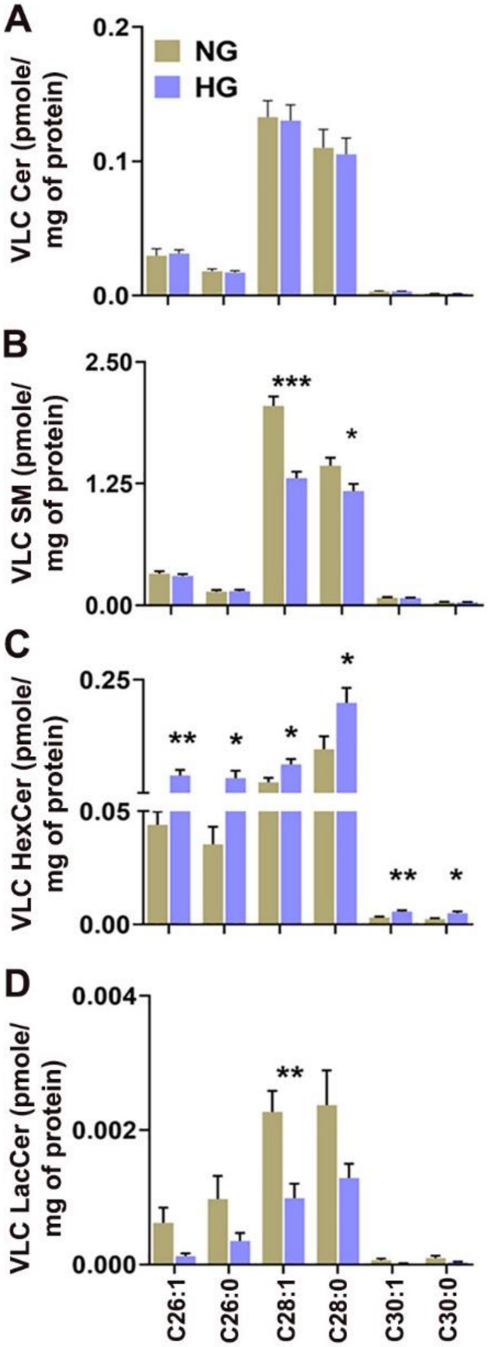

Figure S3: Analysis of different Very Long Chain (VLC) Sphingolipid classes of high glucose-cultured BV2 cells. Total concentration (pmole/mg protein) of different carbon chain length of VLC Ceramide (VLC Cer) (**A**), VLC Sphingomyelin (VLC SM) (**B**), VLC Hexosyl-Ceramide (VLC HexCer) (**C**), VLC Lactosyl-Ceramide (VLC LacCer) (**D**) in normal glucose (NG) and high glucose (HG) cultured cells. *$p < 0.05$, **$p < 0.01$, ***$p < 0.001$, Mean ± SEM, n=6.

Supplementary Table S1: Primers used and their sequences for HREC (Human origin)

Name of Genes	Sequences
B4GALT5	Forward: CCAGAGGGTGACACAGGAAA<br>Reverse: GGCGTCGTATGTGATGTTTG
B4GALT6	Forward: AAGTGGGCTGACAGTGGAAC<br>Reverse: CTGACGCTCCTTGGAATACC
CERS2	Forward: TCTATATCACGCTGCCCCTG<br>Reverse: CTTGCCACTGGTCAGGTAGA
CERS4	Forward: GCATTGAGGACTTTCCTTACCT<br>Reverse: ACCACTCGTTGAAACTGGAC
ELOVL4	Forward: TCCAGAAATATCTTTGGTGG<br>Reverse: GTTAAGGCCCAGTTCAATT
SMPD1	Forward: GAAGAGCTGGAGCTGGAATTA<br>Reverse: CTGGGTCAGATTCAGGATGTAG
SMPD2	Forward: CTT ACC CAG CAC ATC TAC ACT C<br>Reverse: AGC ACG TAG TCA ATG CGG ACA
SPHK1	Forward: AGCACCGATAAGGAGCTGAA<br>Reverse: AGATTTCAGCCTCAGCCAAA
SPHK2	Forward: TTTCCGGAAGAAAGGGATCT<br>Reverse: TAGGCTGGTAGGAGCAAGGA
SPTLC1	Forward: AGGGATTCTGATCCTCTGGATA<br>Reverse: GTTGCCACTCTTCAATCAGTTC
SPTLC2	Forward: TATGGAGCTGGAGTGTGCAG<br>Reverse: GCCAACAAGAGCAGGAATGT
ICAM1	Forward: ATTCCCAGCAGACTCCAATG<br>Reverse: GGCTGCTACCACAGTGATGA
PECAM1	Forward: TGAGGGTGAAGGTGATAGCC<br>Reverse: GGGTTTGCCCTCTTTTTCTC
VEGFA	Forward: ATCTTCAAGCCGTCCTGTGT<br>Reverse: AAATGCTTTCTCCGCTCTGA
IL6	Forward: ACTCACCTCTTCAGAACGAA<br>Reverse: GAAGCATCCATCTTTTTCAG
TNF α	Forward: CTTCTGGCTCAAAAAGAGAA<br>Reverse: GTCAGGGATCAAAGCTGTAG
IL18	Forward: AATAAAGATGGCTGCTGAAC<br>Reverse: CCTTGGTCAATGAAGAGAAC
RPL19	Forward: TCACAGCCTGTACCTGAAGG<br>Reverse: TCGTGCTTCCTTGGTCTTAG
GAPDH	Forward: TGAAGGTCGGAGTCAAGGG<br>Reverse: AGAGTTAAAAGCAGCCCTGGTG

Supplementary Table S2: Primers used and their sequences for BV2 (Rodent origin)

Name of the Genes	Sequence (5′-3′)
CerS2	Forward: GAACGACTATGGCTGCCTGT<br>Reverse: AGGGGTGTTGCCACATAAAG
CerS4	Forward: TGGTTACCACCCAATGTCAC<br>Reverse: TGGACACTGCTTCATCCTGA
Gcs	Forward: GCCATGCAAAACTCTGGTTC<br>Reverse: TGGACACCCCTGAGTTGAAT
Sptlc1	Forward: TTTCCGGTTTAAAAGTGGTG<br>Reverse: CTGATGCTTGGAGGAGGAAG
Sptlc2	Forward: CATTGAGTCCAGAGCCAGAT<br>Reverse: ACACACTGTCCTGGGAGGAA
Sphk1	Forward: GATGCATGAGGTGGTGAATG<br>Reverse: AACAGCAGTGTGCAGTTGAT
Sphk2	Forward: GAAGGCATTGTCACTGTGTC<br>Reverse: GCAGAGAAGAAGCGAGCAGT
Smpd1	Forward: CAGTTCTTTGGCCACACTCA<br>Reverse: TCCGGGGTAGTTTCCATCTA
Smpd2	Forward: ACGTGCTTTACAAGGCAGTC<br>Reverse: AGGACACACAGCAACACCAG
Icam1	Forward: TCAAACGGGAGATGAATGGT<br>Reverse: AGTTTTAGGGCCTCCTCCTG
Pecam1	Forward: ATGACCCAGCAACATTCACA<br>Reverse: TCGACAGGATGGAAATCACA
Vegfa	Forward: ATCTTCAAGCCGTCCTGTGT<br>Reverse: AAATGCTTTCTCCGCTCTGA
IL-6	Forward: TGTGCAATGGCAATTCTGAT<br>Reverse: GGAAGTTGGGGTAGGAAGGA
IL-18	Forward: CAGCCTGTGTTCGAGGATATG<br>Reverse: TCACAGCCAGTCCTCTTACT
Tnf-α	Forward: CTCAAAACTCGAGTGACAAGC<br>Reverse: GTGGGTGAGGAGCACGTAGT
Rpl19	Forward: TCACAGCCTGTACCTGAAGG<br>Reverse: TCGTGCTTCCTTGGTCTTAG
Gapdh	Forward: CTGAACGGGAAGCTCACTG<br>Reverse: ACCACCCTGTTGCTGTAGC
